# Hepatoprotective Effect of Wheat-Based Solid-State Fermented *Antrodia cinnamomea* in Carbon Tetrachloride-Induced Liver Injury in Rat

**DOI:** 10.1371/journal.pone.0153087

**Published:** 2016-04-05

**Authors:** Huan-Wen Chiu, Kuo-Feng Hua

**Affiliations:** 1 Department of Biotechnology and Animal Science, National Ilan University, Ilan, Taiwan; 2 Department of Pathology, Tri-Service General Hospital, National Defense Medical Center, Taipei, Taiwan; IDIBAPS - Hospital Clinic de Barcelona, SPAIN

## Abstract

*Antrodia cinnamomea* (*A*. *cinnamomea*) is an indigenous medical fungus in Taiwan and has multiple biological functions, including hepatoprotective and immune-modulatory effects. Currently, the commercially available *A*. *cinnamomea* are mainly liquid- and solid-state fermented *A*. *cinnamomea*. However, the hepatoprotective effect of solid-state fermented *A*. *cinnamomea* has never been reported. Here we evaluate the ability of air-dried, ground and non-extracted wheat-based solid-state fermented *A*. *cinnamomea* (WFAC) to protect against carbon tetrachloride (CCl_4_)-induced hepatic injury in vivo. The results showed that oral administration of WFAC dose dependently (180, 540 and 1080 mg/kg) ameliorated the increase in plasma aspartate aminotransferase and alanine aminotransferase levels caused by chronic repeated CCl_4_ intoxication in rats. WFAC significantly reduced the CCl_4_-induced increase in hepatic lipid peroxidation levels and hydroxyproline contents, as well as reducing the spleen weight and water content of the liver. WFAC also restored the hepatic soluble protein synthesis and plasma albumin concentration in CCl_4_-intoxicated rats, but it did not affect the activities of superoxide dismutase, catalase, or glutathione peroxidase. In addition, a hepatic morphological analysis showed that the hepatic fibrosis and necrosis induced by CCl_4_ were significantly ameliorated by WFAC. Furthermore, the body weights of control rats and WFAC-administered rats were not significantly different, and no adverse effects were observed in WFAC-administered rats. These results indicate that WFAC is a nontoxic hepatoprotective agent against chronic CCl_4_-induced hepatic injury.

## Introduction

*Antrodia cinnamomea* (*A*. *cinnamomea*; synonym: *Antrodia camphorata*) is an indigenous medicinal fungus in Taiwan. *A*. *cinnamomea* has been demonstrated to possess diverse medicinal and pharmacological activities, particularly its anti-tumor [1–5], anti-inflammatory [6–10], anti-diabetic [11], anti-hypertension [12], anti-bacterial [13], renoprotective [14–16], neuroprotective [17–19], and hepatoprotective effects [20, 21]. Although the greatest number of publications are in regards to the anti-tumor activity of *A*. *cinnamomea*, the Food and Drug Administration has not approved any *A*. *cinnamomea* extracts or purified compounds for clinical anti-tumor applications. Although the therapeutic effects need further investigation, *A*. *cinnamomea* is commonly used as a food supplement and is believed to preserve human vitality and promote longevity [22].

Among the diverse biological functions of *A*. *cinnamomea*, the most recognized function is its hepatoprotective activity [23] because *A*. *cinnamomea* was traditionally used in Taiwan by the aborigines as a traditional prescription for the discomforts caused by drinking alcohol or exhaustion [24]. The fruiting body of *A*. *cinnamomea* protected livers against alcohol-induced liver damage in rats [25, 26] and ameliorated carbon tetrachloride (CCl_4_)-induced hepatic injury in mice [27]. The *A*. *cinnamomea* fruiting body grows very slowly on only the inner cavity of the endemic species *Cinnamomum kanehirae* (Bull camphor tree) Hayata (Lauraceae) in Taiwan. The Taiwanese government has protected *Cinnamomum kanehirae* from forest-denudation because this tree species is relatively rare in nature. Field gathering of the *A*. *cinnamomea* fruiting body is also prohibited [24]. Therefore, the *A*. *cinnamomea* fruiting body is expensive because of its limited availability. Currently, most of the commercially available *A*. *cinnamomea* products come from the submerged liquid or solid-state mycelia cultures. Extracts of mycelium from *A*. *cinnamomea* in submerged liquid culture protect against alcohol-induced liver injury in vitro [28] and in vivo [29]. In addition, fermented filtrates from *A*. *cinnamomea* in submerged liquid culture protect against CCl_4_-induced hepatic toxicity in rats [30]. However, there are as yet no published reports demonstrating the hepatoprotective activity of solid-state cultured *A*. *cinnamomea*.

In the present study, we examined the hepatoprotective activity of wheat-based solid-state fermented *A*. *cinnamomea* (WFAC) in chronic CCl_4_-induced liver injury in rats. In particular, to mimic our regular consumption of *A*. *cinnamomea* products, we fed the rats with air-dried and ground WFAC but not with the extracted WFAC.

## Materials and Methods

### Chemicals

Silymarin, carboxymethyl cellulose, trichloroacetic acid, σ-phthalaldehyde, thiobarbituric acid, p-dimethylaminobenzoaldehyde, n-butanol, pyridine, catalase, hematoxylin and eosin, Sirius red and other chemicals were purchased from Sigma (St. Louis, MO, USA). The protein assay kit was purchased from Bio-Rad Laboratories Inc. (CA, USA)

### Preparation of WFAC

**Fig 1:**
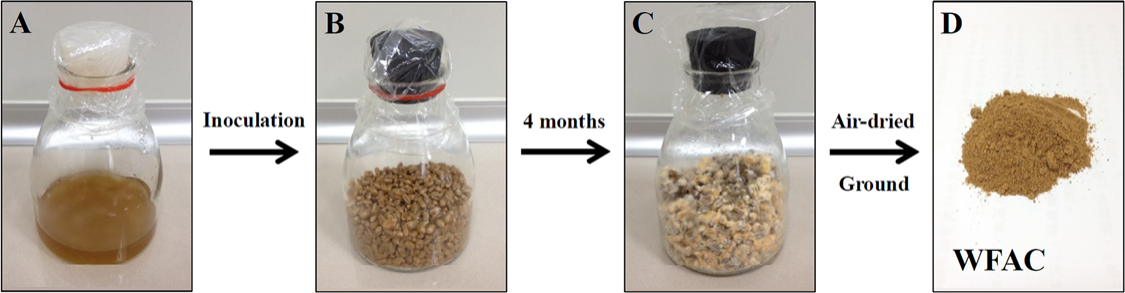
Preparation of WFAC. (A) Liquid fermented *A*. *cinnamomea*. (B) Sterilized wheat before *A*. *cinnamomea* inoculation. (C) 4 months fermented *A*. *cinnamomea* in wheat. (D) Ground WFAC.

The WFAC was obtained from Ruei Sen Biotech Co., Ltd (Taipei, Taiwan). Briefly, WFAC was prepared by inoculating 10 ml of liquid fermented *A*. *cinnamomea* with 100 g of sterilized wheat pre-mixed with 100 ml of medium (2% sugar, 0.5% malt extract, 0.5% yeast extract in ddH_2_O) for 4 months at 25 ± 2°C. Air-dried WFAC was ground and stored at 4°C before feeding it to the rats (Fig 1).

### Ethics statement

All animal manipulations were performed in the Laboratory Animal Center of National Ilan University (Ilan, Taiwan) in accordance with the National Ilan University guide for the care and use of laboratory animals. The procedures used were approved by the Animal Care and Use Committee of the National Ilan University (Approval number: 102–7). All manipulations were performed under isoflurane anesthesia, and all efforts were made to minimize suffering.

### CCl_4_-induced liver injury in rats

Male Wistar rats were obtained from BioLASCO Taiwan Co., Ltd and allowed free access to a standard laboratory chow and tap water ad libitum. The animals were maintained under a 12-h light/dark cycle in an air-conditioned room at 22 ± 2°C. When the rats reached 250–300 g, they were used for the experiments. Rats were divided randomly into six groups: (1) normal control (PBS); (2) vehicle (0.5% carboxymethyl cellulose) plus CCl_4_ treatment; (3) 180 mg/kg WFAC plus CCl_4_ treatment; (4) 540 mg/kg WFAC plus CCl_4_ treatment; (5) 1080 mg/kg WFAC plus CCl_4_ treatment; and (6) 200 mg/kg silymarin plus CCl_4_ treatment. Liver injury was induced in rats by an intraperitoneally (i.p.) administration of 2 ml/kg of 20% CCl_4_ (diluted in olive oil) twice a week for 8 weeks [31]. WFAC-treated rats were given oral administrations of 180, 540, or 1080 mg/kg WFAC, respectively, every day for 8 weeks (started at one day before the administration of CCl_4_). Normal control and CCl_4_-treated rats were treated with PBS or 0.5% carboxymethyl cellulose every day for 8 weeks. The time interval between CCl_4_ and WFAC administrations was 5 h to avoid disturbing the absorption of each other. Blood samples were collected from the tail vein at the end of the first, third, sixth and eighth weeks. Spleens and livers were obtained immediately after the rats were sacrificed at the end of the eighth week, and the weights of the livers and spleens were measured.

### Analyses of plasma aspartate aminotransferase (AST), alanine aminotransferase (ALT) and albumin

AST and ALT activities in the plasma were measured using AST and ALT kits according to the manufacturer’s instructions (Roche Applied Science, Mannheim, DE). The plasma albumin concentration was determined using an ELISA kit (Abcam, Cambridge, MA).

### Analyses of hepatic glutathione (GSH) and lipid peroxidation levels

For the hepatic GSH analysis, 0.5 g of liver tissue was homogenized in 5 ml 1.15% KCl buffer and mixed with 5 ml 10% trichloroacetic acid. After centrifugation at 3000 g for 15 min, 0.01 ml supernatant was mixed with 0.18 ml phosphate-EDTA and 0.01 ml σ-phthalaldehyde (1 mg/ml methanol). After 15 min, the fluorescence at 350 nm excitation and 420 nm emission was read against a blank that contained deionized water to replace the liver tissue. The concentration of GSH was determined using a GSH standard to replace the liver tissue. The results were expressed as μmol/g tissue. For the hepatic lipid peroxidation level analysis, 0.5 g of liver tissue was homogenized in 5 ml 1.15% KCl buffer. After centrifugation at 4,000 rpm for 5 min at 4°C, the supernatant was mixed with the same volume of thiobarbituric acid (0.5% [w/v] thiobarbituric acid in 50% [v/v] acetic acid). Samples were boiled and extracted with n-butanol/pyridine (15:1) buffer and centrifuged for 10 min at 10,000 rpm. The butanol layer containing the thiobarbituric acid-reactive substances (malonaldehyde, MDA) was read at 532 nm. The results are expressed as nmol MDA/mg protein.

### Analysis of hepatic superoxide dismutase (SOD) activity

The method of Xia et al was followed with slight modifications [32]. The SOD activity was measured using the SOD activity assay kit according to the manufacturer’s instructions (RANDOX Lab. Ltd. UK). The method of the SOD activity assay employs xanthine and xanthine oxidase to generate superoxide radicals which react with 2-(4-iodophenyl)-3-(4-nitrophenol)-5-phenyltetrazolium chloride to form a red formazan dye. The superoxide dismutase activity is then measured by the degree of inhibition of this reaction. One unit of SOD is that which causes a 50% inhibition of the rate of reduction of 2-(4-iodophenyl)-3-(4-nitrophenol)-5-phenyltetrazolium chloride under the conditions of the assay. The specific activity of SOD is expressed as U/mg protein.

### Analysis of hepatic catalase activity

The method of Aebi et al was followed with slight modifications [33]. 0.5 g of liver tissue was homogenized in 5 ml 1.15% KCl buffer and centrifuged at 10,000 rpm for 10 min at 4°C. Then the supernatant (5 μl) was mixed with 995 μl of a 30 mM H_2_O_2_ solution prepared in potassium phosphate buffer. Commercially available catalase was used as a standard. A change in the absorbance was monitored at 240 nm for 25 seconds. The specific activity of catalase is expressed as *K*/mg protein. The following equation was generated to calculate the rate constant (*K*): *K* = (2.3/∆t) x log (A1/A2). ∆t: the reaction time interval; A1: the absorbance at 0 second; A2: the absorbance at 25 second.

### Analysis of hepatic glutathione peroxidase (GSH-Px) activity

The method of Xia et al was followed with slight modifications [32]. The GSH-Px activity was measured using the Ransel GSH-Px activity assay kit according to the manufacturer’s instructions (RANDOX Lab. Ltd. UK). GSH-Px catalyzes the oxidation of GSH by cumene hydroperoxide. In the presence of glutathione reductase and NADPH the oxidized glutathione is immediately converted to the reduced form with a concomitant oxidation of NADPH to NADP+. The decrease in absorbance at 340 nm is measured. The specific activity of GSH-Px is expressed as mU/mg protein.

### Analysis of hepatic soluble protein levels and hydroxyproline contents

Protein contents of liver homogenates were determined by the Bio-Rad method using bovine serum albumin as a standard. The results are expressed as mg/g tissue. For hydroxyproline contents, dried liver tissue after hydrolysis was oxidized by H_2_O_2_ and colored by p-dimethylaminobenzoaldehyde, and the absorbance was determined at 540 nm. The amount of hydroxyproline is expressed as μg/g tissue. The percentage of water content of liver was determined by (dry weight/wet weight) x 100%.

### Liver histopathology

Rats were sacrificed at the end of the eighth week. The livers were immediately fixed in a 10% buffered formalin phosphate solution, embedded in paraffin, cut into 4–5 μm thick sections, and stained with hematoxylin and eosin and Sirius red. Liver fibrosis and necrosis were graded according to the guidelines of hepatoprotective assessment of the Food and Drug Administration, Ministry of Health and Welfare, Taiwan.

### Index compounds of *A*. *cinnamomea* in WFAC

Eight signature triterpenoids compounds of *A*. *cinnamomea* in WFAC were determined and quantitated by quantitative LC-MS/MS. The data was provided by ABM International Lab Inc. (Pingtung, Taiwan).

### Statistical analysis

Data are presented as the mean ± SD. All other experimental data, except the pathological findings, were analyzed using a one-way ANOVA with Dunnett’s test. Liver histopathological examination data were analyzed by the Kruskall-Wallis non-parametric test followed by a Mann-Whitney U-test.

## Results

### WFAC reduces plasma AST and ALT formation and increases the plasma albumin concentration in CCl_4_-intoxicated rats

**Fig 2:**
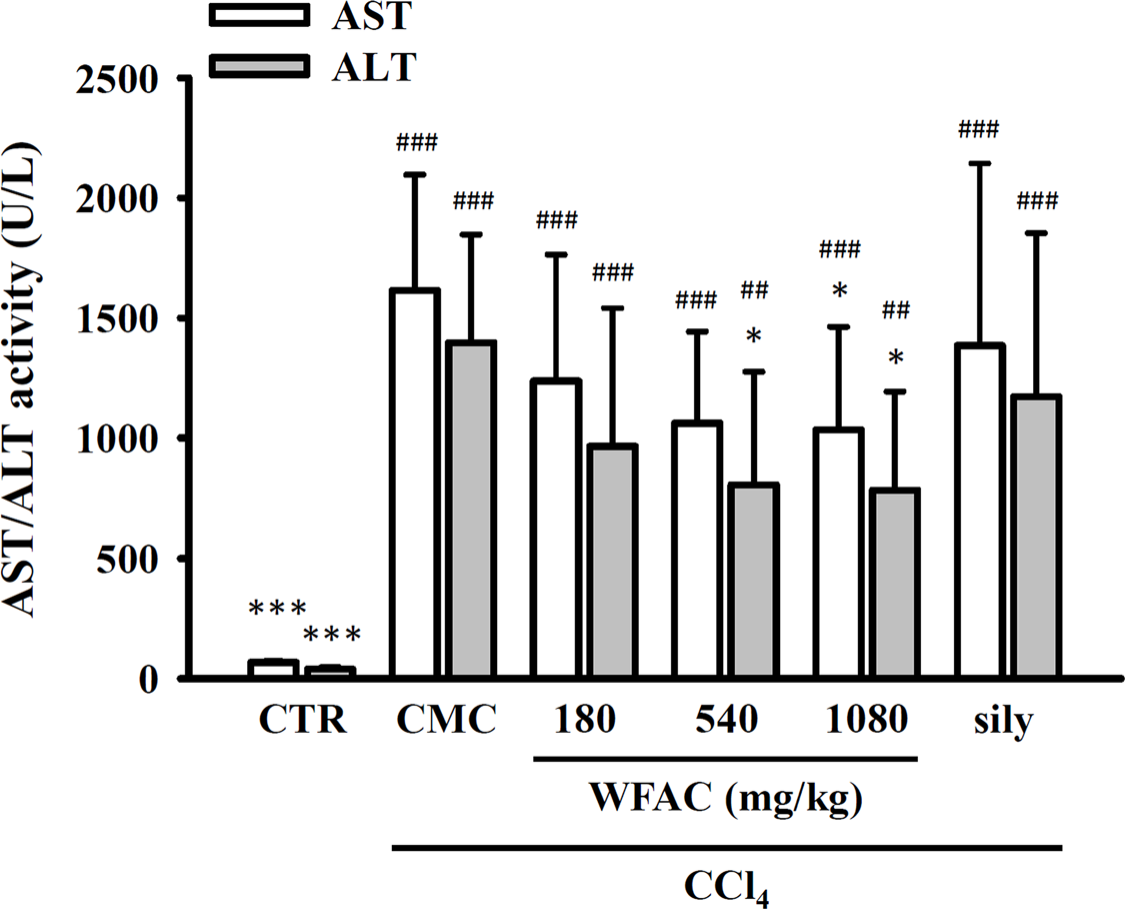
Effect of WFAC on plasma AST and ALT activities in CCl_4_-intoxicated rat. Blood samples collected at the end of the eighth weeks after CCl_4_ intoxication. All values are the means ± S.D. (n = 10). ## *p* < 0.01 and ### *p* < 0.001 compared with the control group. * *p* <0.05 and *** *p* <0.001 compared with the CCl_4_ + CMC group. CTR: control; Sily: silymarin (200 mg/kg).

**Fig 3:**
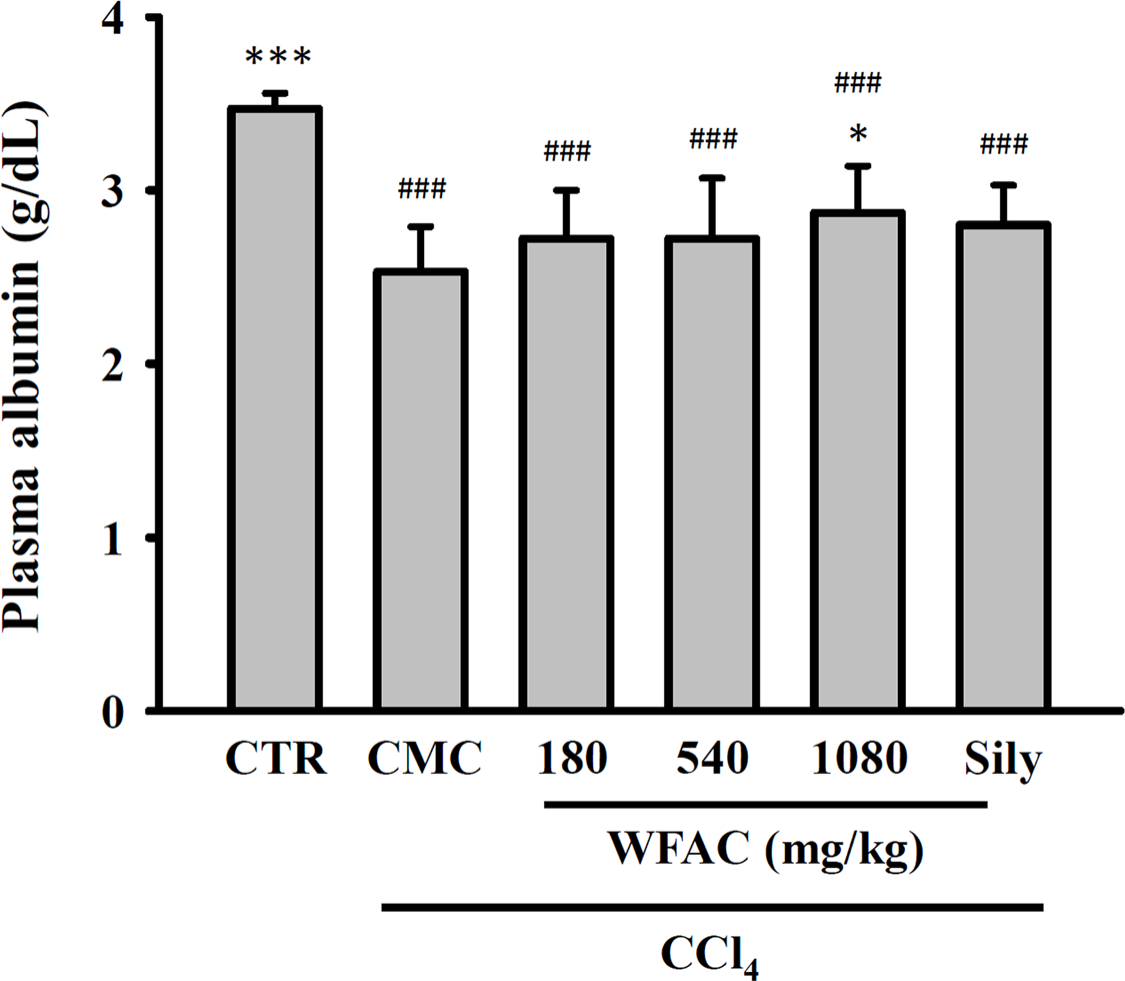
Effect of WFAC on plasma albumin concentration in CCl_4_-intoxicated rat. Blood samples collected at the end of the eighth weeks after CCl_4_ intoxication. All values are the means ± S.D. (n = 10). ### *p* < 0.001 compared with the control group. * *p* <0.05 and *** *p* <0.001 compared with the CCl_4_ + CMC group. CTR: control; Sily: silymarin (200 mg/kg).

As shown in Fig 2, the plasma levels of AST and ALT were elevated by the oral administration of CCl_4_ at the end of the first week and then increased to 1615.0 ± 482.0 and 1396.7 ± 450.8 U/L by the eighth week, respectively. In the control group (PBS treatment), plasma AST and ALT levels were 70.5 ± 8.3 and 39.4 ± 7.9 U/L, at the end of the eighth weeks, respectively. Oral administration of WFAC (180, 540 and 1080 mg/kg) reduced plasma AST and ALT levels dose dependently at the end of the first to eighth weeks. The WFAC (1080 mg/kg) markedly reduced plasma AST and ALT levels to 1034.3 ± 429.0 and 784.0 ± 409.6 at the end of the eighth week in CCl_4_-intoxicated rats. However, oral administration of silymarin (200 mg/kg) also reduced plasma ALT and AST levels in CCl_4_-intoxicated rats, but it did not reach statistical significance. Albumin is a protein made by the liver, and lower-than-normal levels of plasma albumin may be a sign of liver injury. The plasma albumin concentration was significantly reduced by CCl_4_ intoxication at the end of the eighth week, and WFAC (1080 mg/kg) significantly increased the plasma albumin concentration (Fig 3). Again, silymarin (200 mg/kg) increased the plasma albumin concentration in CCl_4_-intoxicated rats, but it did not reach statistical significance (Fig 3). Additionally, the changes in body weights of the CCl_4_-intoxicated or WFAC-treated rats were similar to those of the control rats, although the body weights of the CCl_4_-intoxicated rats at the end of the first and sixth weeks was temporarily reduced (Table 1). These results suggested that the administration of WFAC did not have any adverse effects.

**Table 1:** Effect of WFAC on body weights in CCl_4_-intoxicated rat.

	Body weight (g)									
Drugs	Doses (mg/kg)	Before CCl_4_	Week 1	Week 2	Week 3	Week 4	Week 5	Week 6	Week 7	Week 8
Control	‒	265.1 ± 10.9	293.9 ± 23.5	314.6 ± 28.1	327.5 ± 25.2	350.5 ± 17.1	371.5 ± 19.2	388.4 ± 16.6	408.8 ± 18.4	416.5 ± 25.2
CCl_4_ + CMC	‒	265.0 ± 9.0	266.4 ± 17.1^#^	305.8 ± 20.0	316.7 ± 18.5	330.5 ± 26.9	351.9 ± 23.6	357.5 ± 10.7#	380.9 ± 22.0	389.7 ± 20.9
CCl_4_ + WFAC	180	267.5 ± 11.7	274.0 ± 16.2	317.7 ± 23.2	326.8 ± 27.6	344.5 ± 30.6	358.9 ± 13.0	363.9 ± 36.6	388.3 ± 38.4	399.2 ± 35.0
CCl_4_ + WFAC	540	272.0 ± 13.8	282.1 ± 13.4	324.0 ± 19.4	333.5 ± 19.1	343.4 ± 17.1	362.6 ± 14.9	359.0 ± 20.8	391.8 ± 14.0	404.5 ± 20.3
CCl_4_ + WFAC	1080	268.6 ± 8.4	271.1 ± 12.2	310.4 ± 21.1	322.0 ± 27.4	330.0 ± 36.0	343.9 ± 36.3	347.3 ± 35.1	365.8 ± 35.0	384.5 ± 28.3
CCl_4_ + silymarin	200	265.9 ± 7.8	265.6 ± 16.3	292.0 ± 33.8	303.1 ± 33.8	338.3 ± 26.3	353.1 ± 26.0	359.4 ± 24.8	378.6 ± 25.4	394.6 ± 24.5

All values are the means ± S.D. (n = 10).

# *p* < 0.05 compared with the control group.

### WFAC reduces hepatic lipid peroxidation levels in CCl_4_-intoxicated rats

**Fig 4:**
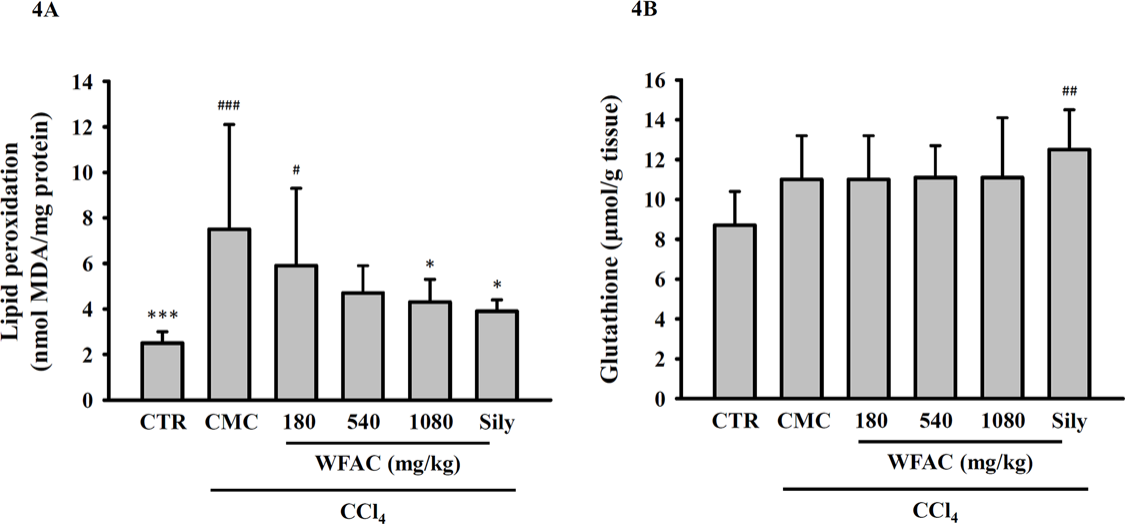
Effect of WFAC on the hepatic levels of lipid peroxidation and glutathione in CCl_4_-intoxicated rat. Liver tissue samples collected at the end of the eighth weeks after CCl_4_ intoxication. (A) Levels of lipid peroxidation. (B) Levels of glutathione. All values are the means ± S.D. (n = 10). # *p* < 0.05, ## *p* < 0.01 and ### *p* < 0.001 compared with the control group. * *p* <0.05 and *** *p* <0.001 compared with the CCl_4_ + CMC group. CTR: control; Sily: silymarin (200 mg/kg).

**Fig 5:**
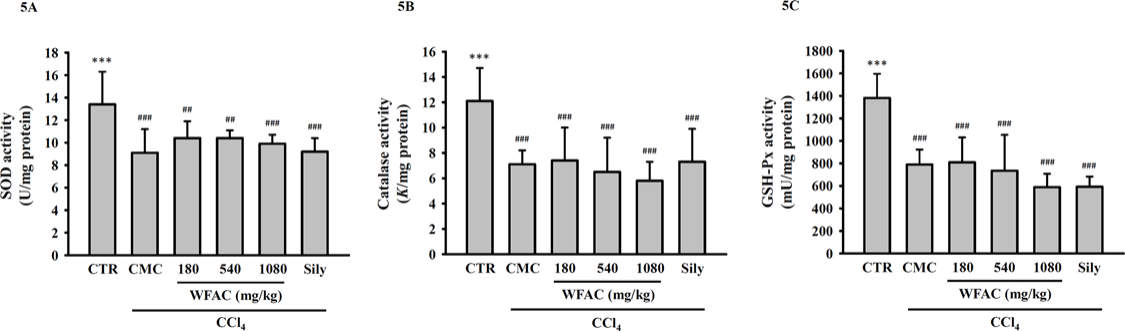
Effect of WFAC on hepatic SOD, catalase and GSH-Px activities in CCl_4_-intoxicated rat. Liver tissue samples collected at the end of the eighth weeks after CCl_4_ intoxication. (A) SOD activity. (B) Catalase activity. (C) GSH-Px activity. All values are the means ± S.D. (n = 10). ## *p* < 0.01 and ### *p* < 0.001 compared with the control group. *** *p* <0.001 compared with the CCl_4_ + CMC group. CTR: control; Sily: silymarin (200 mg/kg).

Lipid peroxidation is one of the consequences of uncontrolled oxidative stress in organ injuries caused by oxidative damage. As shown in Fig 4A, CCl_4_ intoxication increased the level of hepatic reactive aldehyde malondialdehyde (MDA), a marker of lipid peroxidation, at the end of the eighth week. The WFAC (1080 mg/kg) and silymarin (200 mg/kg) significantly reduced the hepatic MDA level in the CCl_4_-intoxicated rats. In addition to plasma MDA levels, plasma glutathione levels have also been used as a determinate of oxidative status. However, CCl_4_ intoxication and WFAC administration did not affect the hepatic glutathione levels (Fig 4B). Furthermore, although CCl_4_ intoxication reduced the activities of hepatic anti-oxidant enzymes, such as SOD, catalase, and GSH-Px, WFAC and silymarin did not restore the levels of these enzymes (Fig 5A–5C).

### WFAC increases hepatic soluble protein levels and reduces hepatic hydroxyproline contents in CCl_4_-intoxicated rats

**Fig 6:**
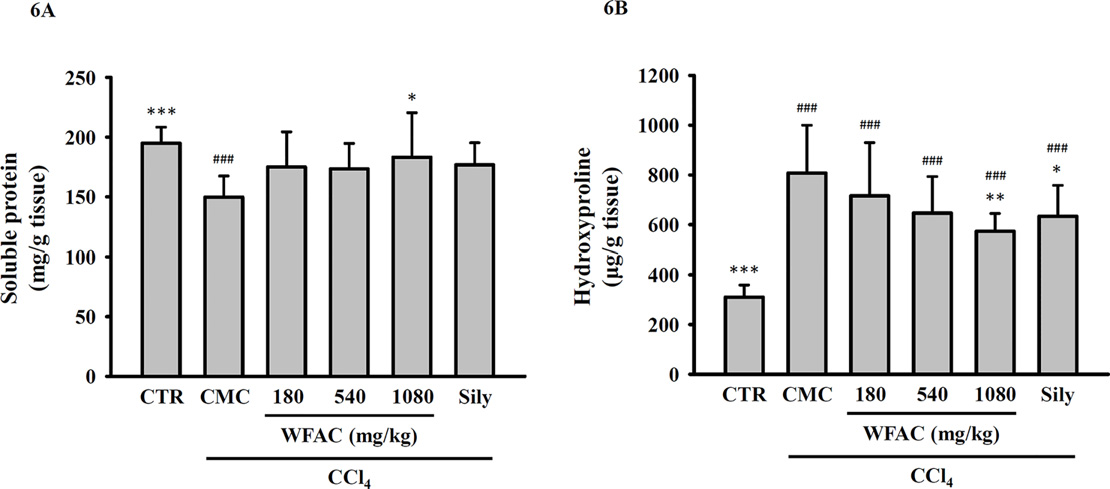
Effect of WFAC on hepatic soluble protein levels and hydroxyproline contents in CCl_4_-intoxicated rat. Liver tissue samples collected at the end of the eighth weeks after CCl_4_ intoxication. (A) Soluble protein levels. (B) Hydroxyproline contents. All values are the means ± S.D. (n = 10). ### *p* < 0.001 compared with the control group. * *p* <0.05, ** *p* <0.01 and *** *p* <0.001 compared with the CCl_4_ + CMC group. CTR: control; Sily: silymarin (200 mg/kg).

The hepatic soluble protein levels will be reduced in liver injury. We found that the hepatic soluble protein levels (mg/g tissue) significantly decreased from 194.8 ± 13.5 in control rats to 149.7 ± 17.7 CCl_4_ in CCl_4_-intoxicated rats at the end of the eighth week. WFAC (1080 mg/kg) significantly increased the hepatic soluble protein levels to 183.1 ± 37.3 (mg/g tissue) in CCl_4_-intoxicated rats. Silymarin (200 mg/kg) also increased the hepatic soluble protein, but it did not reach statistical significance (Fig 6A). In addition, we asked whether WFAC ameliorated the liver fibrosis induced by CCl_4_ intoxication. To investigate liver fibrosis, the total collagen present in the liver was determined by estimating the hydroxyproline content, a product of collagen metabolism. We found that the hydroxyproline content (μg/g protein) increased from 309.9 ± 49.0 in control rats to 807.3 ± 192.5 in CCl_4_-intoxicated rats. WFAC (1080 mg/kg) significantly decreased the hydroxyproline content to 572.8 ± 72.0 (μg/g protein) in CCl_4_-intoxicated rats. Silymarin (200 mg/kg) also decreased the hydroxyproline content, but it did not reach statistical significance (Fig 6B).

### WFAC reduces spleen weight and liver water content in CCl_4_-intoxicated rats

**Fig 7:**
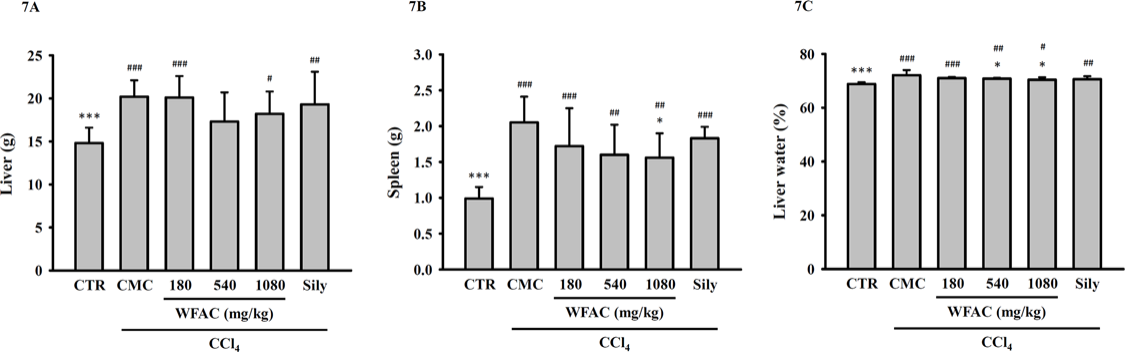
Effect of WFAC on the weights of the liver and spleen and the water content of livers in CCl_4_-intoxicated rat. Liver tissue samples collected at the end of the eighth weeks after CCl_4_ intoxication. (A) Liver. (B) Spleen. (C) Liver water content. All values are the means ± S.D. (n = 10). # *p* < 0.05, ## *p* < 0.01 and ### *p* < 0.001 compared with the control group. * *p* <0.05 and *** *p* <0.001 compared with the CCl_4_ + CMC group. CTR: control; Sily: silymarin (200 mg/kg).

As shown in Fig 7, In control rats, the weight of the liver (Fig 7A) and spleen (Fig 7B) were 14.8 ± 1.8 and 0.99 ± 0.16 g, respectively. The weight of the liver and spleen of CCl_4_-intoxicated rats increased to 20.2 ± 1.9 and 2.05 ± 0.36 g, respectively, at the end of the eighth week. WFAC (1080 mg/kg) significantly decreased the spleen weight to 1.56 ± 0.34 g in CCl_4_-intoxicated rats. However, WFAC at 180 and 540 mg/kg did not decrease the spleen weight. WFAC and silymarin did not decrease the liver weight in CCl_4_-intoxicated rats. In addition, as shown in Fig 7C, the water content of the liver increased from 68.8 ± 0.7% in control rats to 72.1 ± 1.9% in CCl_4_-intoxicated rats. WFAC at 540 and 1080 mg/kg significantly reduced the water content of the liver to 70.8 ± 0.3 and 70.4 ± 0.9%, respectively, in CCl_4_-intoxicated rats. Silymarin reduced the water content of the liver to 70.6 ± 1.1, but it did not reach statistical significance.

### WFAC reduces liver fibrosis and necrosis in CCl_4_-intoxicated rats

**Fig 8:**
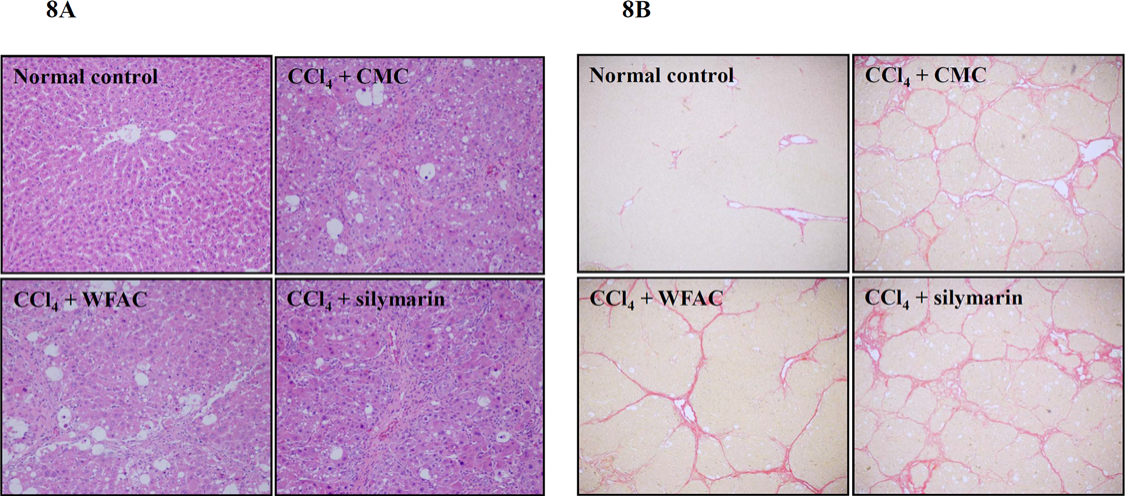
Hematoxylin/eosin and Sirius red staining showing the effect of the WFAC on hepatic histopathology in CCl_4_-intoxicated rat. Liver tissue samples collected at the end of the eighth weeks after CCl_4_ intoxication. (A) Hematoxylin and eosin staining of a representative liver tissue. (B) Sirius red staining of a representative liver tissue.

Histopathological changes of necrotic and lipid-laden hepatocytes of liver sections were assessed at the end of the eighth week after CCl_4_ intoxication. The typical intense centrilobular necrosis of hepatotoxicity and inflammatory cell infiltration were observed in CCl_4_-intoxicated rats (Fig 8A). Marked macro- and microvesicular fatty changes of hepatocytes around the central vein and parenchymal disarrangement were also found in CCl_4_-intoxicated rats (Fig 8A). WFAC administration (1080 mg/kg) improved these pathological changes in the liver tissue (Fig 8A**)**. In addition, Sirius-red staining was performed to detect hepatic fibrosis. In the CCl_4_-intoxicated rat, the liver showed fibrous connective tissue proliferation and fiber interval formation, which was associated with a disorder of the lobular structure in the portal area. Most rat livers appeared to have pseudo lobules (Fig 8B). In the WFAC administered rats (1080 mg/kg), livers appeared to have fibrous connective tissue proliferation, the formation of a few fiber intervals in the portal area, and the occasional pseudo lobule (Fig 8B). Scoring of these histopathological changes showed that WFAC administration (1080 mg/kg) significantly reduced the liver necrosis and fibrosis, but silymarin did not (Table 2).

**Table 2:** Effect of WFAC on hepatic histopathological changes in CCl_4_-intoxicated rat.

		Fibrosis					Necrosis				
Drugs	Doses (mg/kg)	−	+	++	+++	++++	−	+	++	+++	++++
Control	‒	10	0	0	0	0	10	0	0	0	0
CCl_4_ + CMC	‒	0	0	4	5	1	0	1	3	4	2
CCl_4_ + WFAC	180	0	2	4	3	1	0	2	3	4	1
CCl_4_ + WFAC	540	0	4	3	2	1	0	2	4	3	1
CCl_4_ + WFAC	1080	0	3	5	2	0	0	4	4	1	1
CCl_4_ + silymarin	200	0	1	4	4	1	0	1	4	4	1

Grade designation of the histological findings; (−) normal, (+) very slight, (++) slight, (+++) moderate, (++++) severe. Each value is the number of animals with grading changes.

* *p* <0.05 compared with the CCl_4_ + CMC group for fibrosis.

# *p* < 0.05 compared with the CCl_4_ + CMC group for necrosis.

### Index compounds of A. cinnamomea in WFAC

Eight signature triterpenoids compounds of *A*. *cinnamomea* in WFAC were determined and quantitated by LC-MS/MS as shown in Table 3. The compounds could be divided into two chemical groups: (1) triterpenoids and steroids [dehydroeburicoic acid (1.43 ppm), dehydrosulfurenic acid (75.64 ppm), antcin A (1.68 ppm), antcin B (1.00 ppm), antcin C (0.84 ppm), antcin H (non-detectable), antcin K (2.70 ppm)]; (2) benzenoids [1,4-dimethoxy-2,3-methylenedioxy-5-methylbenzene (2747 ppm)].

**Table 3:** Index compounds of *A*. *cinnamomea* in WFAC.

Compound name	Content^1^ (ppm)	Bioactivities
1,4-dimethoxy-2,3-methylenedioxy-5-methylbenzene	2747	anti-tumor [54]
Dehydroeburicoic acid	1.43	anti-diabetic [53]; hepatoprotective [47]; anti-inflammation [8]; anti-tumor [52]
Dehydrosulphurenic acid	75.64	anti-tumor [55]
Antcin A	1.68	anti-inflammation [57]
Antcin B	1.00	anti-tumor [55]
Antcin C	0.84	anti-oxidant and Hepatoprotective [43]
Antcin H	N. D.^2^	
Antcin K	2.70	anti-tumor [56]

^1^Eight signature triterpenoids compounds of *A*. *cinnamomea* in WFAC were quantitated by quantitative LC-MS/MS.

^2^N. D. means non-detectable.

## Discussion

*A*. *cinnamomea* is a unique and endemic mushroom of Taiwan and is one of the most popular heath foods used in Asia, particularly in Taiwan. *A*. *cinnamomea* has been well-known for its medicinal properties, particularly with regard to liver complaints [20]. However, the *A*. *cinnamomea* fruiting body is expensive, therefore, its application is limited. Currently, most of the commercially available *A*. *cinnamomea* comes from artificial cultures, such as submerged liquid and solid-state mycelia cultures. In this study, we investigated the hepatoprotective effect of *A*. *cinnamomea* obtained by wheat-based solid-state fermentation. For an appropriate conversion of drug doses from humans to rats, we converted the human dose to an animal dose based on the body surface area, and not by a simple conversion based on body weight, because body surface area correlates well with several physiological parameters in mammals, including blood volume, circulating plasma proteins, basal metabolism, oxygen utilization, caloric expenditure, and renal function [33]. In this study, the animal dose for the rat was calculated by considering the suggested daily usage doses of solid-state fermented *A*. *cinnamomea* (2 g/ day in human). The dose calculation was performed on the basis of body surface area using a conversion factor of 0.018 (200 g rat/70 kg human) [34]. Therefore, rats were administered 180 mg/ kg WFAC, which is equal to 2 g/70 kg in humans. However, 180 mg/kg WFAC was ineffective in preventing liver cirrhosis induced by CCl_4_ in rats. To get significant hepatoprotective effect, WFAC requires a dose at 1080 mg/kg. It should be noted that WFAC is an un-extracted raw material and is mainly composed of wheat. Although the concentration of signature triterpenoids compounds of *A*. *cinnamomea* in WFAC is not so high, WFAC is a cost-efficiency way of producing these rare bioactive compounds. These results provide the scientific evidence that human daily usage dose of WFAC should be increased to get the significant hepatoprotective effect. Otherwise, partial purification of these rare bioactive compounds from WFAC is needed to reduce the daily usage dose of WFAC.

AST and ALT are enzymes found mainly in the liver, and they are released into the bloodstream when the liver is diseased or damaged, causing the levels of the enzymes in the plasma to rise. Therefore, the amount of AST and ALT in the plasma is directly related to the extent of the liver damage. We found that administering WFAC significantly reduced the plasma AST and ALT levels in CCl_4_-intoxicated rats, indicating that WFAC is able to ameliorate CCl_4_-induced liver injury. Although plasma AST and ALT levels are a valuable aid primarily in the diagnosis of liver disease, they are not specific for liver disease because AST and ALT are also found in red blood cells, heart cells, muscle tissue and other organs, such as the pancreas and the kidneys [35]. To further confirm the hepatoprotective effect of WFAC, the plasma albumin concentration in CCl_4_-intoxicated rats was measured. Albumin is the major plasma protein produced in the liver that circulates in the bloodstream. Albumin is essential for maintaining the oncotic pressure in the vascular system. Decreased plasma albumin levels indicate chronic liver failure caused mainly by cirrhosis, a late stage of hepatic fibrosis that results in widespread distortion of the normal hepatic architecture [36]. Our data showed that decreased plasma albumin levels were observed at the end of the sixth and eight weeks after CCl_4_ intoxication, and these effects were partially recovered in WFAC-treated rats. In addition, WFAC also increased the CCl_4_-induced decrease in hepatic soluble protein levels. These results indicated that WFAC improved the liver function in CCl_4_-intoxicated rats, including increasing protein synthesis.

Cirrhosis or liver fibrosis lead to an abnormally high blood pressure in the portal vein called portal hypertension. The spleen becomes enlarged as the increased pressure interferes with blood flow from the spleen into the portal blood vessels [37]. CCl_4_ intoxication caused spleen enlargement in rats, and this effect was inhibited by WFAC treatment. This result indicates that WFAC administration ameliorated portal hypertension in CCl_4_-intoxicated rats. CCl_4_ intoxication causes liver damage and inflammation, and liver regeneration can occur in rats, which increases liver weight [38]. In this study, we found that CCl_4_ intoxication increased the liver weight and water content of the liver in rats. Although the liver weight was not affected by WFAC treatment, the water content of the liver was significantly reduced. These results suggest that WFAC reduced the inflammation in the liver. Liver fibrosis is a wound response to severe liver damage and occurs in many forms of chronic liver damage, including CCl_4_ intoxication [39]. Hydroxyproline is detected specifically in collagen, which plays a major role in liver fibrosis and appears to reflect the degree of liver fibrosis [40]. WFAC treatment decreased the CCl_4_-induced increase in liver hydroxyproline levels, indicating that WFAC ameliorated liver fibrosis. The anti-fibrosis effect of WFAC was also confirmed by histopathological staining with Sirius-red. It has been demonstrated that CCl_4_-induced liver fibrosis occurs via free radical-mediated lipid peroxidation [41]. CCl_4_ intoxication increased hepatic lipid peroxidation, and this effect was reduced by WFAC, indicating that oxidative stress in the liver was reduced. Although CCl_4_ intoxication reduced the activities of hepatic antioxidant enzymes, including SOD, catalase, and GSH-Px, WFAC did not restore the activities of these enzymes. The level of glutathione, an important antioxidant in the liver, was also not changed by WFAC. It has been suggested that liver injury caused by CCl_4_ through lipid peroxidation and protein oxidation, which involves cytochrome P450-dependent formation of trichloromethyl free radicals and ROS [42]. It has been showed that antcin C from *A*. *cinnamome*a protect liver cells from oxidative stress and cell death via Nrf2/ARE activation [43]. We suggested that WFAC reduced the lipid peroxidation level in the CCl_4_-intoxicated rats might through inducing Nfr2/ARE activation or other anti-oxidative pathways; however, the detailed mechanism needs further investigation.

Although both the fruiting body and fermented mycelia of *A*. *cinnamomea* have been demonstrated to exhibit hepatoprotective activity in various hepatic injury models [27, 44, 45], the bioactive components are not fully understand. Antrodin B, a maleimide derivative isolated from the fruiting body of *A*. *cinnamomea*, inhibited transforming growth factor-β1-induced fibrosis in hepatic stellate cells [46]. Antcin C (from the fruiting body) and antroquinonol (from mycelium) protect hepatic cells from free radical- and ethanol-induced oxidative stress, respectively, through Nrf-2 activation [28, 43]. In addition, eburicoic acid and dehydroeburicoic acid isolated from the fruiting body of *A*. *cinnamomea* were identified as hepatoprotective compounds in CCl_4_-intoxicated mice [47]. Furthermore, a neutral polysaccharide isolated from the mycelium of *A*. *cinnamomea* ameliorated *Propionibacterium acnes* and lipopolysaccharide induced hepatic injury in mice [48]. *A*. *cinnamomea* polysaccharides also have been demonstrated to exhibit anti-hepatitis B virus effects and immune modulation properties [49–51]. WFAC contains Antcin C and dehydroeburicoic acid, which might be response for its hepatoprotective effect [43, 47]. Dehydroeburicoic acid not only exhibits hepatoprotective effect but also shows anti-inflammation [8], anti-tumor [52] and antidiabetic activities [53]. WFAC also contains 1,4-dimethoxy-2,3-methylenedioxy-5-methylbenzene, dehydrosulphurenic acid, antcin B and antcin K, which exhibit anti-tumor activities [54–57]. In addition, anti-inflammatory compound antcin A in WFAC might contribute to its hepatoprotective effect [58].

In this study, we used silymarin as a positive control for ameliorating liver injury. However, silymarin only reduced the CCl_4_-induced increase in lipid peroxidation and had no effect on other liver injury markers. Some clinical review papers show that silymarin was not able to improve the mortality, histopathological changes, or liver function indexes in patients with chronic liver disease [59, 60]. Although there are many papers showing that silymarin ameliorates chronic liver injury in animal models, some papers indicate that silymarin is useless for chronic liver injury [61–63]. These results may come from the low bioavailability of silymarin [64]. Therefore, Letteron et al used high dose silymarin (800 mg/kg i.p.) to prevent CCl_4_-induced lipid peroxidation and hepatotoxicity in mice [65]. In this study, we used silymarin purchased from different companies and also increased the dose to 500 mg/kg. However, silymarin did not have a significant effect in our experimental model.

In conclusion, we demonstrated that WFAC has several benefits in CCl_4_-intoxicated rats, including: 1) reducing plasma AST and ALT levels; 2) increasing plasma albumin and hepatic soluble protein levels; 3) reducing spleen weight and water content of the liver; 4) and reducing lipid peroxidation and fibrosis of liver. This is the first report to show that the raw material of heat-based solid-state fermented *A*. *cinnamomea* protects against CCl_4_-induced hepatic injury in vivo.
